# Flow Sorting Enrichment and Nanopore Sequencing of Chromosome 1 From a Chinese Individual

**DOI:** 10.3389/fgene.2019.01315

**Published:** 2020-01-09

**Authors:** Lukas F. K. Kuderna, Manuel Solís-Moruno, Laura Batlle-Masó, Eva Julià, Esther Lizano, Roger Anglada, Erika Ramírez, Alex Bote, Marc Tormo, Tomàs Marquès-Bonet, Òscar Fornas, Ferran Casals

**Affiliations:** ^1^ Institut de Biologia Evolutiva, CSIC-Universitat Pompeu Fabra, Parc de Recerca Biomèdica de Barcelona (PRBB)", Barcelona, Spain; ^2^ Genomics Core Facility, Departament de Ciències Experimentals i de la Salut, Universitat Pompeu Fabra, Parc de Recerca Biomèdica de Barcelona (PRBB), Barcelona, Spain; ^3^ Serveis Científico-Tècnics, Institut Hospital del Mar d'Investigacions Mèdiques (IMIM), Barcelona, Spain; ^4^ Flow Cytometry Unit, Centre for Genomic Regulation (CRG), The Barcelona Institute for Science and Technology (BIST), Barcelona, Spain; ^5^ Scientific IT Core Facility, Departament de Ciències Experimentals i de la Salut, Universitat Pompeu Fabra, Parc de Recerca Biomèdica de Barcelona (PRBB), Barcelona, Spain; ^6^ CNAG-CRG, Centre for Genomic Regulation (CRG), The Barcelona Institute of Science and Technology, Barcelona, Spain; ^7^ Departament de Ciències Experimentals i de la Salut, Universitat Pompeu Fabra (UPF), Barcelona, Spain; ^8^ Institució Catalana de Recerca i Estudis Avançats (ICREA), Barcelona, Spain; ^9^ Institut Català de Paleontologia Miquel Crusafont, Universitat Autònoma de Barcelona, Barcelona, Spain; ^10^ Flow Cytometry Unit, Departament de Ciències Experimentals i de la Salut, Universitat Pompeu Fabra (UPF), Parc de Recerca Biomèdica de Barcelona (PRBB), Barcelona, Spain

**Keywords:** chromosome enrichment, nanopore sequencing, chromosome sequencing, chromosome sorting, flow karyotyping, structural variation, genome assembly

## Abstract

Sorting of individual chromosomes by Flow Cytometry (flow-sorting) is an enrichment method to potentially simplify genome assembly by isolating chromosomes from the context of the genome. We have recently developed a workflow to sequence native, unamplified DNA and applied it to the smallest human chromosome, the Y chromosome. Here, we modify improve upon that workflow to increase DNA recovery from chromosome sorting as well as sequencing yield. We apply it to sequence and assemble the largest human chromosome - chromosome 1 - of a Chinese individual using a single Oxford Nanopore MinION flow cell. We generate a selective and highly continuous assembly whose continuity reaches into the order of magnitude of the human reference GRCh38. We then use this assembly to call candidate structural variants against the reference and find 685 putative novel SV candidates. We propose this workflow as a potential solution to assemble structurally complex chromosomes, or the study of very large plant or animal genomes that might challenge traditional assembly strategies.

## Introduction

Structural genetic variation is abundant and has important functional impact (Conrad et al., 2010). A human genome has been estimated to harbor more than 2,000 structural variants (SV), which are typically defined as variants that affect at least 50 bp (Mills et al., 2011). They include balanced (inversions, translocations) and unbalanced forms (insertions, deletions, duplications) (Mills et al., 2011). The functional impacts are mainly produced by altering the number of copies of coding sequences, and thus the gene expression levels, or disrupting coding or regulatory regions with a potential effect not only in the closer genes but also extending to hundreds of kilobases away (Weischenfeldt et al., 2013). SVs have been associated both to Mendelian and common disorders, although it is difficult to exactly define the phenotypic impact due to the presence of many functional regions in the same variant, as well as variable expressivity and penetrance even across family (Weischenfeldt et al., 2013). Nevertheless, and despite of its high abundance and potential impact, the study of SVs has been less accelerated in comparison to single nucleotide variants and short insertions and deletions (indels). It has been mainly limited by the short reads generated by massive parallel sequencing technologies and the relatively low coverage in large sequencing efforts (e.g., 1,000 Genomes Project) (Huddleston and Eichler, 2016). Also, determining the exact position and mechanism of origin of SVs is not straightforward often due to the presence of terminal repetitive sequences and recurrence, and can be especially challenging in complex structural variants with more than two breakpoints and overlapping or nested rearrangements (Collins et al., 2017; Stephens et al., 2018). All this makes difficult the generation of systematic catalogues of SVs and the estimation of allelic population frequencies.

The recently emerging possibility to obtain reads of up to of several Megabases in length on the Oxford Nanopore platform represents an important advance for the study of structural variants and genome assembly, as it greatly simplifies them (Giordano et al., 2017; Payne et al., 2019). These sequencing technologies can be combined with enrichment strategies, from capture by hybridization to Cas9 based methodologies, to restrict the analysis to specific regions also increasing the sequencing yield. Chromosome isolation is an alternative enrichment strategy which will better maintain molecular integrity with the potential of generating longer sequence reads (Jiang et al., 2015; Kozarewa et al., 2015; Gabrieli et al., 2018).

We recently developed a workflow to isolate and sequence native flow-sorted human Y chromosomes on an Oxford Nanopore MinION device (Kuderna et al., 2019). We sought to apply this method to other chromosomes to generate a population specific long read assembly, namely for a chromosome 1 of a Chinese individual. We show the generalizability and improve the protocol in terms of DNA recovery and sequencing yield.

## Method

### Chromosome Isolation and Sequencing

Chromosome preparation, staining, sorting, DNA purification, concentration and sequencing were performed as previously described in (Kuderna et al., 2019) with some modifications (see supplementary methods). Briefly, chromosomes were prepared from a lymphoblastoid cell line derived from a Chinese individual (Coriell, cat. no. HG00542) by using a polyamine isolation method. Modifications: hypotonic solution was incubated at 37°C for 20 min and polyamine isolation buffer was incubated on ice for 30 min. Additionally, potassium citrate was replaced by sodium citrate and sodium sulfite to a final concentration of 10 and 25 mM respectively and incubated at least 2 hours to enhance peak resolution in the flow karyotype. Purification and concentration were carried out as previously described with the exception that after SPRI bead purification DNA was eluted in 20 µl of Low TE buffer. Libraries for sequencing were prepared from the purified DNA following the protocol of the Ligation Sequencing Kit SQK-LSK 109 (Oxford Nanopore Technologies). A 48 hours MinION run was performed in a FLO-MIN106 flow cell.

### Assembly, Error Correction, and SV Calling

We called bases from the raw fast5 signal data using Guppy (v. 2.2.2) with the following command line:

guppy_basecaller -i $input -s $output –flowcell FLO-MIN106 –kit SQK-LSK109 -t 4 –disable_pings



We mapped the base called reads onto GRCh38 using Minimap2 (Li, 2018) with the ont preset. We sorted the mappings and converted them to bam using samtools (Li et al., 2009):
minimap2 -x map-ont -t8 -a hg38.fa basecalled_reads.fq| samtools sort -@8 -O BAM - -o mapped_reads.bam


We unsuccessfully tried to assemble the raw reads into contigs using canu (v. 1.8) (Koren et al., 2017) with default parameters assuming a “genome” size of 250 Mb. This command used more than 15 Tb of disk space and did not finish to yield a successful assembly on our systems. To overcome the issue, we extracted mappings on chromosome 1 and assembled only those:
canu -p HG00542-chr1 -d HG00542-chr1 genomeSize = 250m -nanopore-raw basecalled_reads.chr1_mappings.fq


We corrected errors in the resulting contigs with Nanopolish (v. 0.11.0) (Simpson et al., 2017). To this end, we remapped the raw reads to the assembly as shown above. We then went on to create a read db with nanopolish, and split the assembly into chunks of 500 Kb with nanopolish_makerange.py and called the variants of each chunk with nanopolish variants
nanopolish_makerange.py –segment-length 500000 –overlap-length 1000 HG00542- HG00542-chr1.contigs.fasta | xargs -i echo nanopolish variants –ploidy 2 –consensus -o {}.consensus.round1.vcf -w {} -r basecalled_reads.fq -b HG00542- HG00542-chr1.contigs.self-mappings.bam -g HG00542-chr1.contigs.fasta | sh


We then incorporated the corrections into the assembly:
nanopolish vcf2fasta -g HG00542-chr1.contigs.fasta *vcf.


We aligned the resulted polished assembly to GRCh38 chr1 with MUMmer (Kurtz et al., 2004)
nucmer -maxmatch -l 100 -c 500 hg38.chr1.toplevel.fa. HG00542-chr1.contigs.polished.fasta -prefix HG00542.polished.r1.vs.hg38_chr1


We fed the resulting alignments to Assemblytics to obtain candidates for SV Assemblytics HG00542.polished.r1.vs.hg38_chr1.delta HG00542.polished.r1.vs.hg38_chr1.10kanchor.50kmax 10000 bin/Assemblytics/


We generated an additional callset with Sniffles (v. 1.0.8) (default parameters), using the previously mapped reads from minimap2. For downstream analysis we only retained calls annotated as “precise” by Sniffles:sniffles -m mapped_reads.bam -v sniffles_callset.vcf.


We filtered all calls that fall within 2 Mb of distance to the centromere or telomeres.

### Comparative Repeat Annotation

We ran repeatmasker (v. 4.0.7) with the same parameters on both our assembly and GRCh38 to create comparable annotations:
RepeatMasker -e ncbi -pa 8 -s -species human -no_is -noisy -dir. -a -gff -u assembly.fa


We calculated the divergence of each repeat to its consensus using the “calcDivergenceFromAlign.pl” utility included in the RepeatMasker package.

## Results and Discussion

**Figure 1:**
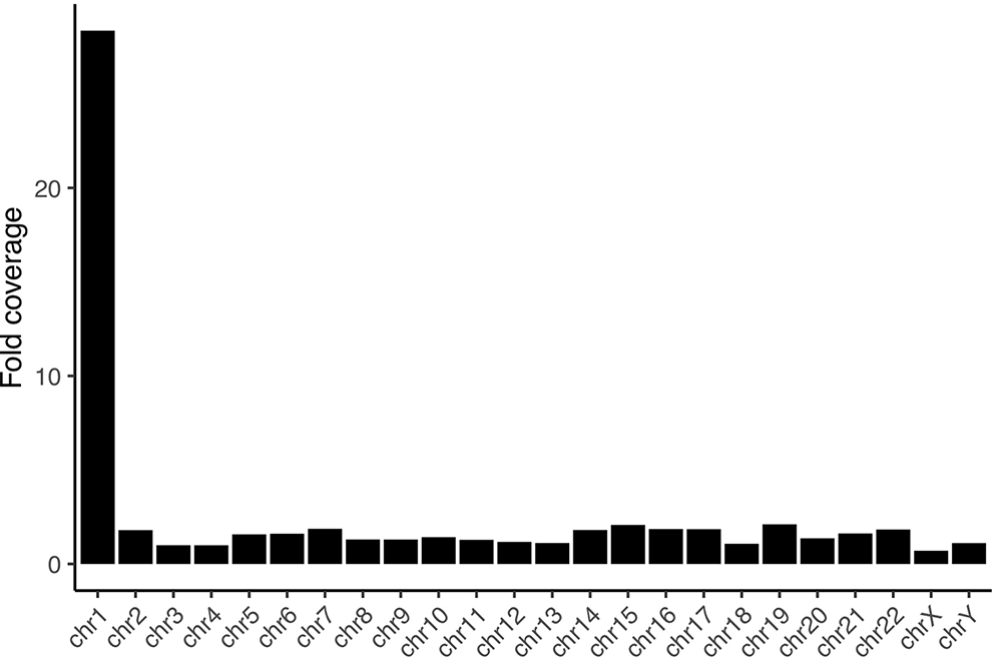
Fold coverage per chromosome. The sequencing is selective for chromosome 1, with all other chromosomes being depleted from the data.

We sorted 10 million individual chromosomes 1 from a lymphoblastoid cell line derived from a Chinese individual (HG00542) to obtain 5 μg of DNA from a total of 205x10^6^ cultured cells from six independent experiments (see **Supplementary Figure 1**). Of that, we used 2 million sorted chromosomes theoretically corresponding to 1μg of DNA (Gribble et al., 2009). From this material, we constructed a library using an Oxford Nanopore ligation kit and ran a single MinION flow cell on it. Given limitations in DNA recovery from flow-sorted material we have previously encountered, we have made adjustments to the sorting and purification protocol (see methods and supplementary methods). The higher DNA recovery and higher loading amount on the flow cell yielded between 20 and 131 times more data from a single run than our previous efforts, meaning that a single flow cell is now sufficient to assemble the largest human chromosomes after flow-sorting it. These differences are likely also attributable to improvements in the pore chemistry and base-calling algorithms. After base calling with Guppy, we were left with 2.5 million reads summing to 14.3 Gb of data with a read length N50 of 15.4 Kb. Of them, 10.6 Gb mapped readily to GRCh38, and 5.6 Gb to chromosome 1 (see **Supplementary Figure 2**). The average coverage on chromosome 1 was 28.4 fold, the coverage on the remaining chromosomes ranged from 0.7 fold on chrX to 2.1 fold on chr19 (**Figure 1**). This amounts to an 8-fold enrichment over a random sampling from bases along a diploid male genome (see **Supplementary Table 5**). All other chromosomes are depleted from the data, with depletion ranging from 0.27 fold on chr4 to 0.61 fold on chrY. We find the average depletion on non-target chromosomes to be more efficient in this dataset compared to our previous effort on the Y chromosome (0.42 fold versus 0.61 fold). Nevertheless, we observe the enrichment on the target chromosome to be less efficient compared to the Y chromosome. This fact is likely attributable to the more challenging physical separation of chromosome 1 in a human flow karyogram, as the chromosome clusters are not as well defined as e.g. the one of chrY (**Supplementary Figure 1**).

**Figure 2:**
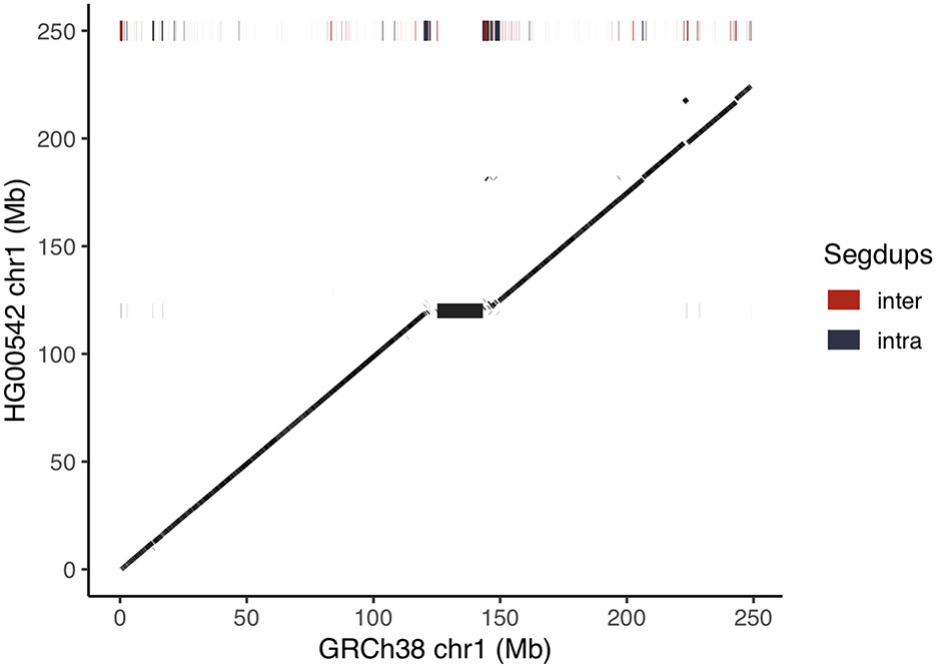
Dot-plot of HG00542 assembly versus GRCh38 chromosome 1. The chromosomes are laid out on the respective axis and a dot denotes aligned sequence between the two assemblies. Bars at the height of 250 Mb on the Y axis show the positions of segmental duplications in GRCh38. The assembly is largely colinear to the reference. The large black block in the center of the dot-plot delimits the 18 Mb centromere of chromosome 1.

We assembled the raw data using Canu (Koren et al., 2017). To this end, we removed reads that do not map to GRCh38 chr1 to ease the computational load of the assembly (see Method). While this might confound the assembly in regions of large insertions or translocations, it significantly eases computational burden. We polished remaining single base substitution and indel errors in the resulting assembly with Nanopolish (Simpson et al., 2017). The final assembly has a length of 227.8 Mb and consists of 154 contigs with an N50 of 10.5 Mb. We aligned our assembly to GRCh38 chromosome 1, whose total resolved sequence length (i.e. excluding “N” from the assembly) is 230.5 Mb. We find 98.8% of our assembly to cover 97.6% of the reference (**Figure 2**, **Supplementary Table 4**). The boundaries of our contigs are enriched in segmental duplications and satellite repeats in the reference. We observe the highest degree of fragmentation around the centromeric region, which is littered with satellite repeats and segmental duplications, and therefore particularly challenging to assemble. Contigs mapping to these regions also show a drop off in identity to the reference. The centromere on chromosome 1 of GRCh38 is an 18 Mb long heterochromatic expansion flanked by segmental duplications that is still unresolved, as in most other human chromosomes (Jain et al., 2018).

**Figure 3:**
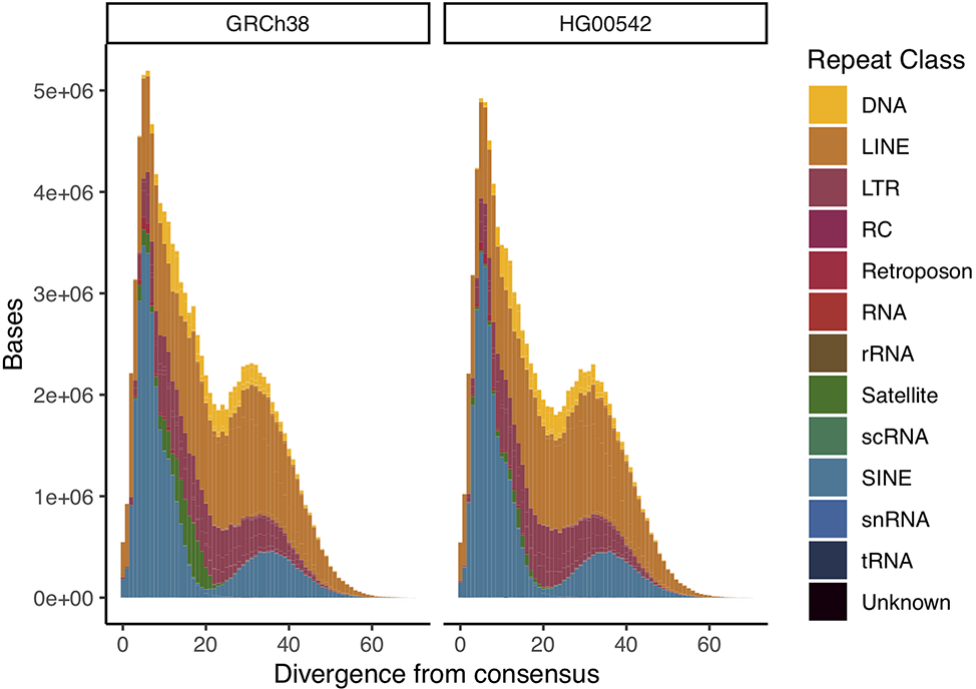
Comparative repeat landscapes of GRCh38 chromosome 1 and HG00542 chromosome 1. We find equal resolution across most repeat classes.

To assess repeat resolution, we produced a comparative repeat annotation between our assembly and GRCh38 using repeatmasker (Smit et al.). We find both assemblies to have very similar proportions annotated as repetitive overall and for all given repeat families. We then calculated the divergence of all annotated repetitive elements to their consensus sequence to create “repeat landscapes”. We find these landscapes to be highly similar between the two assemblies. We measured repeat resolution in our assembly as the proportion of bases annotated as a given repeat type. We find them to be of comparable quality across all major repeat types, with centromeric & telomeric satellite sequences constituting an exception (**Figure 3**, **Supplementary Figure 3**, **Supplementary Table 1**).

**Figure 4:**
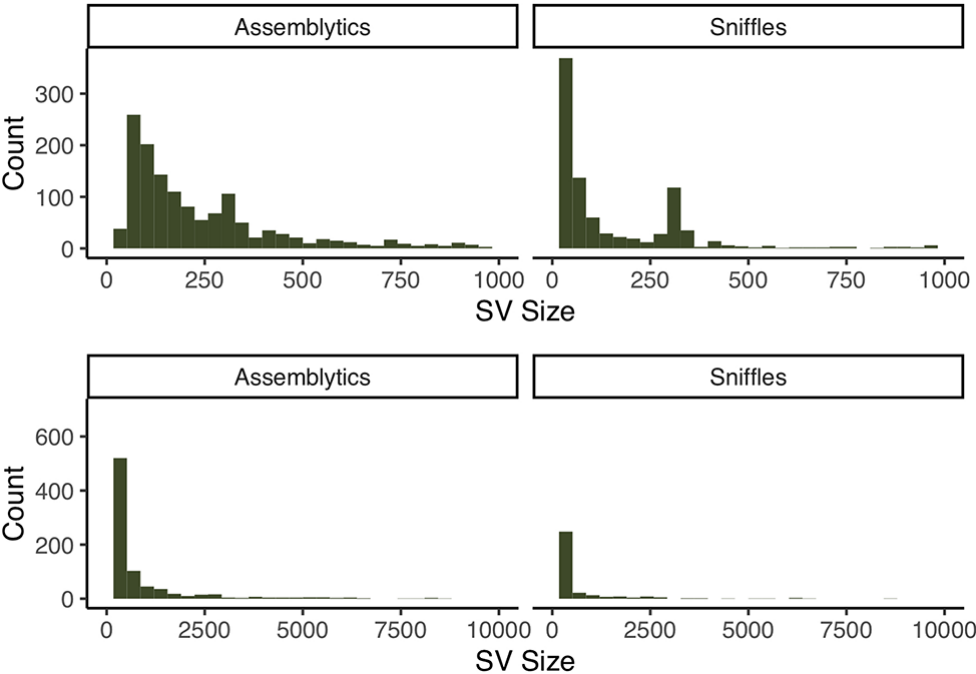
Size distribution of SV calls from both Assemblytics and Sniffles at different resolutions. Both call sets have clear peaks around 300 bp corresponding to Alu-elements.

We then used the assembly to generate a call set of candidate structural variants (SVs) against GRCh38. To this end, we aligned our assembly to the reference using MUMmer (Kurtz et al., 2004) and looked for patterns of SVs using Assemblytics (Nattestad and Schatz, 2016). We additionally ran an orthogonal detection approach by mapping the raw reads to GRCh38 and running Sniffles (Sedlazeck et al., 2018). To minimize erroneous calls, we excluded putative SVs within 2 Mb of the centromeric and telomeric regions, as the higher degree of segmental duplications and assembly fragmentation is more likely to yield false positive calls (Audano et al., 2019). By this means, we identified 1,325 SVs with Assemblytics and 940 with Sniffles along chromosome 1. We find 405 of the calls to intersect between the two sets, with 61.4% and 56.9% to be unique to Assemblytics and Sniffles, respectively (see **Supplementary Figures 4–8**). Of the intersecting calls, we find 230 to lie within genic regions, and 8 to affect the coding portions of the gene (**Figure 4** and **Supplementary Tables 2–3, 6–7**).

**Figure 5:**
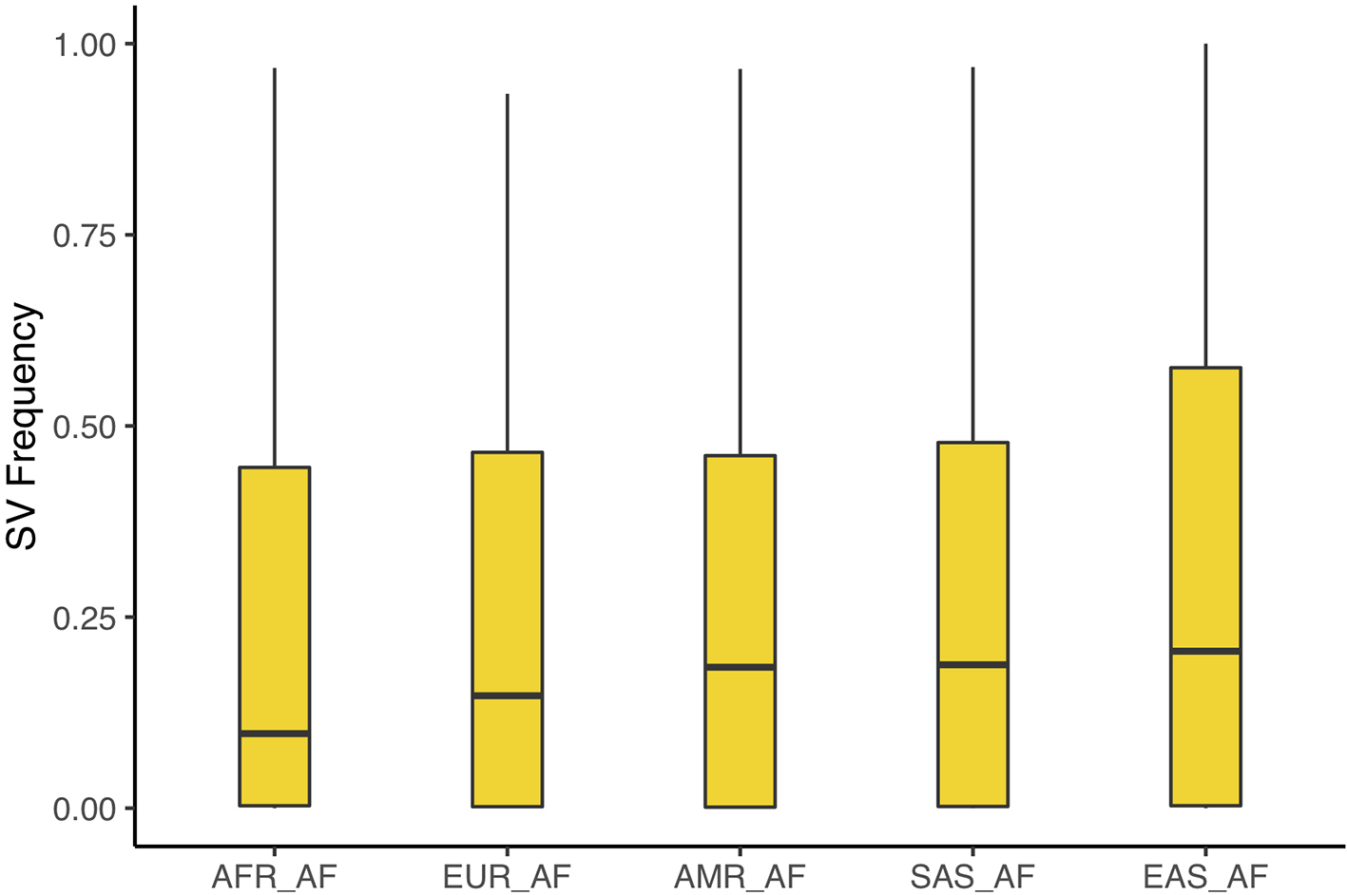
Population frequencies of SV discovered in our assembly that overlap calls by the 1,000 genomes project. We find these SVs to be most frequent in East Asian populations, followed South Asian and American, European, and lastly African populations.

We sought to assess novel SVs on the one hand, and population frequencies of SVs that might have previously been described in other datasets. To this end, we contrasted our calls against those generated by the 1,000 genomes consortium (Sudmant et al., 2015), which used short-read data to detect SVs with several different algorithms. This study detected 4,653 SVs on chr1 among 2,504 individuals. Unsurprising, given the technological differences between the two datasets, we find comparatively little overlap between the two call sets with 466 SVs that overlap over 40% in either of them. We calculated the frequencies of overlapping SVs in each of the superpopulations of the 1,000 genomes data. After removing variants with an allele count of 2 or less, and multiallelic positions we find these SVs to reach the highest frequencies in east Asian populations (20.5%); South Asian and American populations exhibit similar frequencies (18.8% and 18.4%) followed by European (14.7%) and lastly African (9.8%) populations (see **Figure 5** and **Supplementary Figures 9–12**). We additionally contrasted our calls with more recently generated ones that also used long-read assemblies for detection (Audano et al., 2019). Among 15 individuals included in that study there are 6,646 SVs along chr1. We find 291 SVs from our call set to overlap those. The calls by Audano et al. include a Chinese individual (HX1) and one from Korea (AK1) (Seo et al., 2016; Shi et al., 2016). Among the overlapping SVs, we find 108 deletions (or 44%) and 32 insertions (64%) to also be present in both these individuals. Lastly, we identify 685 novel SV candidate loci that have not previously been described in neither of the above datasets.

In summary, we generated a highly continuous and selective assembly of the largest human chromosome from a Chinese individual from flow-sorted native DNA. We show that increased efficiency in DNA recovery from flow-sorted chromosomes, as well as improvements in nanopore technology, allow for single chromosome assemblies from a single MinION flow cell and that as little as 28-fold coverage is sufficient to yield an assembly with a contig N50 over 10 Mb. As with previous reports, we still find room for improvement in terms of base accuracy. We observe a deletion bias in our data, which we find to be twice as frequent as insertions. However, given the constant development in both pore design and base calling algorithms, these issues are likely to improve in the near future.

It is worth noting that flow-sorting chromosomes only constitutes a viable approach if the species' chromosomes are sufficiently distinct in terms of size and GC content. As an example, human chromosomes 9–12 have size differences of up to 6%. However, with our approach, they are hardly distinguishable by flow karyotyping because of similar GC-content across them. Conversely, human chromosomes 1–2 have a size difference of only 1.6%. Nevertheless, they differ more strongly in GC content, making them clearly distinguishable by flow karyotyping. Addressing this “sortability” of a species' genome is achieved empirically. While assembling mammalian genomes has become routine, there is still a large amount of plants and animals for which traditional whole-genome shotgun assembly methods might be computationally prohibitive given their massive genome sizes. We expect assembling flow-sorted chromosomes to be a viable alternative in these cases.

## Data Availability Statement

The sequencing data has been deposited at the European Nucleotide Archive (ENA) under the accession PRJEB34445. The assembly can be accessed under GCA_902652775.1.

## Ethics Statement

Ethical review and approval was not required for the study on human participants in accordance with the local legislation and institutional requirements. Written informed consent for participation was not required for this study in accordance with the national legislation and the institutional requirements.

## Author Contributions

LK, MS-M, LB-M, TM-B, OF, and FC conceived the project. LK, MS-M, LB-M, EJ, EL, OF, and FC designed the study. LK, MS-M, LB-M, and MT performed the bioinformatic analysis. EJ, EL, RA, ER, and AB performed the experimental analysis. All the authors participated in the analysis of the data. LK, MS-M, LB-M, EJ, OF, and FC wrote the manuscript.

## Funding

This study was funded by grants RTI2018-096824-B-C22 from the Agencia Estatal de Investigación-Ministerio de Ciencia, Innovación y Universidades (Spain) and FEDER (EU) to OF and FC, SAF2015-68472-C2-2-R from the Ministerio de Economía y Competitividad (Spain) and FEDER (EU) to FC, the Centro de Excelencia Severo Ochoa, and by Direcció General de Recerca, Generalitat de Catalunya (2017SGR-702). TM-B is supported by BFU2017-86471-P (MINECO/FEDER, UE), U01 MH106874 grant, Howard Hughes International Early Career, Obra Social “La Caixa” and Secretaria d'Universitats i Recerca and CERCA Programme del Departament d'Economia i Coneixement de la Generalitat de Catalunya (GRC 2017 SGR 880). LK is supported by an FPI fellowship associated with BFU2014-55090-P (MINECO/FEDER, UE) and by an EMBO Short-Term Fellowship STF-8286. LB-M is supported by a Formació de personal Investigador fellowship from Generalitat de Catalunya (2018_FI_B00072). MS-M is supported by the María de Maetzu Programme (MDM-2014-0370-16-3).

## Conflict of Interest

The authors declare that the research was conducted in the absence of any commercial or financial relationships that could be construed as a potential conflict of interest.
