# Supplementary material for: Flow Sorting Enrichment and Nanopore Sequencing of Chromosome 1 From a Chinese Individual
Source: Front Genet. 2020 Jan 9;10:1315. doi: 10.3389/fgene.2019.01315 (PMC6962354; doi:10.3389/fgene.2019.01315)
Supplement: Supplementary file 1 [file DataSheet_1.docx]

# **Supplementary material**

# **Flow sorting enrichment and nanopore sequencing of chromosome 1 from a Chinese individual**

**Lukas F.K. Kuderna*^1^, Manuel Solís-Moruno*^1,2^, Laura Batlle-Masó*^1,2^, Eva Julià*^3,4^, Esther Lizano*^1^, Roger Anglada^2^, Erika Ramírez^4^, Alex Bote^4^, Marc Tormo^2,5^, Tomàs Marquès-Bonet ^1,6,7,8,9^, Oscar Fornas*^4,7^, Ferran Casals*^2^**

1 Institut de Biologia Evolutiva, (CSIC-Universitat Pompeu Fabra), PRBB, Doctor Aiguader 88, Barcelona, Catalonia 08003, Spain.

2 Genomics Core Facility, Departament de Ciències Experimentals i de la Salut, Universitat Pompeu Fabra, Parc de Recerca Biomèdica de Barcelona, 08003 Barcelona, Spain.

3 Institut Hospital del Mar d’Investigacions Mèdiques (IMIM), Carrer del Doctor Aiguader 88, PRBB Building, Barcelona 08003, Spain.

4 Centre for Genomic Regulation (CRG), The Barcelona Institute for Science and Technology, Carrer del Doctor Aiguader 88, Barcelona 08003, Spain.

5 Scientific IT Core Facility, Departament de Ciències Experimentals i de la Salut, Universitat
Pompeu Fabra, Parc de Recerca Biomèdica de Barcelona, 08003 Barcelona, Spain.

6 CNAG‐CRG, Centre for Genomic Regulation (CRG), The Barcelona Institute of Science and Technology, Baldiri Reixac 4, Barcelona 08028, Spain.

7 Universitat Pompeu Fabra (UPF), Doctor Aiguader 88, Barcelona 08003, Spain.

8 Institució Catalana de Recerca i Estudis Avançats (ICREA), Passeig Lluís Companys 23, Barcelona, Catalonia 08010, Spain.

9 Institut Català de Paleontologia Miquel Crusafont, Universitat Autònoma de Barcelona, Edifici ICTA-ICP, c/ Columnes s/n, Cerdanyola del Vallès, Barcelona 08193, Spain

* These authors contributed equally to this work

Contact: lukas.kuderna@upf.edu, oscar.fornas@upf.edu, ferran.casals@upf.edu

**Supplemental methods**

Chromosome suspension for sorting.

Chromosome suspension samples were prepared as previously described in *Kuderna L et al*. with some modifications using a lymphoblastoid cell line derived from a Chinese individual (Coriell, cat. no. HG00542) by polyamine isolation method as follows. Cells were cultured in RPMI-1640 medium supplemented with 2mM L-glutamine, 15% fetal bovine serum and antibiotics at initial concentration of no <150,000 viable cells per ml. Close to confluence, cells were subcultured to block them in mitosis by adding Colcemid to a final concentration of 0.1 μg ml−1 and incubated for 6–7 h. To swell and stabilize mitotic cells, they were centrifuged 5 min at 300 g at room temperature. Cells were slowly resuspended in 10 ml hypotonic solution (Hypotonic solution: 75mM KCl, 10mM to be MgSO4, 0.2mM spermine, 0.5mM spermidine. pH 8.0) and incubation was modified to 37ºC for 20 min. The swollen cells were centrifuged at 300 g for 5min and the pellet was resuspended in 1.5 ml of ice-cold polyamine isolation buffer (15mM Tris, 2mM EDTA, 0.5mM EGTA, 80mM KCl, 3mM dithiothreitol, 0.25% Triton X-100, 0.2mM spermine, 0.5mM spermidine, pH 8.0) and the incubation was modified by increasing it to 30 minutes and on ice to better release the chromosomes. Pellet was vigorously vortexed for 30 s to liberate the chromosomes from the mitotic cells. The suspension was filtered through a 35 μm mesh filter and stored at 4°C until its sorting. Chromosomes staining was performed with chromomycin-A3 and Hoechst 33258 at a final concentration of 40 μgml−1 and 5 μgml−1 respectively, in presence of divalent cations (10mM MgSO4). Staining was performed for at least 8 h at 4 °C to equilibrate the dyes. Before the sample analysis, to enhance peak resolution in the flow karyotype, sodium citrate and sodium sulphite to a final concentration of 10mM and 25mM respective were added, replacing previously described potassium citrate, and was incubated at least 2 hours.

Instrument setup and sorting

Chromosome sorting was performed on BD Influx cell sorter (Becton Dickinson, San Jose, CA), a jet-in-air cell sorter that was selected for its relatively easy manual daily fine-tuning and high-resolution capabilities. Instrument setup was as previously described in *Kuderna L et al*. Shortly, we used a 100μm nozzle with 20 p.s.i. sheath pressure and piezoelectric frequency at 38.7 KHz. Instrument performance was optimized using 8-peaks Rainbow beads (SpheroTM Rainbow Calibration Particles 3.0–3.4 μm, BD Biosciences), 1-peak UV beads for UV laser alignment (AlignflowTM Flow Cytometry Alignment 2.7 μm, Molecular Probe) and 1-peak 457 nm for deep-blue laser alignment (FluoresbriteTM Plain YG Microspheres 1.0 μm, Polysciences, Inc.). Those beads were respectively used for 488-blue, 355-UV, and 457-deep-blue optimal laser alignment and instrument fine tuning to obtain the highest resolution of chromosome detection and sorting. The threshold for chromosome sorting was set triggering in chromomycin-A3 fluorescence on 457nm laser as primary excitation line and chromomycin-A3 fluorescence was detected at 550/50nm band-pass filter. Hoechst fluorescence was detected at 460/50 BP band-pass filter using UV laser for its excitation. All parameters were collected in lineal mode and analyzed with the BD FACSTM Software (v. 1.0.0.0.650, Becton Dickinson, San Jose, CA). Gating strategy for chromosome sorting was simple because only a bi-parametrical dot-plot Hoechst versus chromomycin-A3 fluorescence was used (supplementary figure 1).

The sorting was performed across 6 independent experiments with the following yields:

Experiment 1: 3 flasks with 8x10e6 cells per flask yielding 1.36 x10e6 sorted chromosome 1

Experiment 2: 3 flasks with 12x10e6 cells per flask yielding 0.96 x10e6 sorted chromosome 1

Experiment 3: 4 flasks with 14 x10e6 cells per flask yielding 3.67 x10e6 sorted chromosome 1

Experiment 4: 4 flasks with 14 x10e6 cells per flask yielding 2.20 x10e6 sorted chromosome 1

Experiment 5: 2 flasks with 4 x10e6 cells per flask yielding 0.40 x10e6 sorted chromosome 1

Experiment 6: 3 flasks with 8.5 x10e6 cells per flask yielding 1.37x10e6 sorted chromosome 1

Total: 19 flasks with a total of 205x10e6 cells yielding 9.96x10e6 sorted chromosome 1

Purification of sorted chromosomes for sequencing.

We sorted ten million individual chromosomes-1 from a lymphoblastoid cell line derived from a Chinese individual (HG00542) obtaining 5μg of DNA from a total of 205x10e6 cultured cells from six independent experiments. Then, only 1μg out of 5μg sorted DNA (theoretically corresponding to 2x10e6 sorted chromosomes as reported by *Gribble et al.*) were treated overnight with proteinase K (20 mg ml−1) at 50 °C overnight. Proteinase K inactivation was performed by incubating at 65ºC during 5 minutes. To remove proteinase K, chromomycin-A3 and Hoechst 33258 samples were dialyzed using a Pur-ALyzer™ Maxi Dialysis column with a molecular weight cut-off of 50 kDa (Sigma- Aldrich) in 2 liters of TE buffer for 48h in continuous agitation, by exchanging the buffer every 10–16 h.

Sample volume was reduced up to a volume of approximately 60μl by evaporation in a miVac DNA concentrator (Barnstead GeneVac, Ipswich, UK). Finally, purification was carried out by pooling the concentrated DNA into two tubes and subjecting it to a solid-phase reversible immobilisation (SPRI) bead purification with a 2X ratio (SPRIbeads/sample), after the purification the sample was eluted into 20μl of TE buffer. Sample concentration was determined by absorbance at 260 nm with a NanoDrop 2000 (Thermo Scientific) and by fluorometric assay with the Qubit 2.0 using the Qubit dsDNA HS kit (Invitrogen) obtaining 22ng/μl (440ng in the whole eluted sample) and 15ng/μl (300ng in the whole eluted sample) respectively. We hypothesize the difference in quantification between the platforms is due to residual intercalating dyes from the sorting experiment, which might interfere with the platforms.

Enrichment calculations

For the enrichment calculation we asked how much more frequent we observe reads on the chromosome one compared to sequencing the whole genome at random (i.e. sequencing a whole genome shotgun). To this end we first calculated the probability s of randomly sampling a base from a given chromosome, accounting for ploidy in a male individual. All chromosomal lengths were corrected for callable sequence by not considering gapped bases (“N”):

$$s=\frac{length chromosome*ploidy}{diploid lenght of genome}$$

We than calculated the empirically observed probability o of sampling a base on a given chromosome from the mappings of our sequencing data:

$$o=\frac{mapped bases on chromosome}{all mapped bases}$$

The enrichment factor f of a given chromosome was then calculated as follows:

$$f=\frac{o}{s}$$

The enrichment factor therefore informs about how much more often (or in the case of non-target chromosome, how much less often) we see a base on a given chromosome samples compared to our expectation of sampling that base at random.


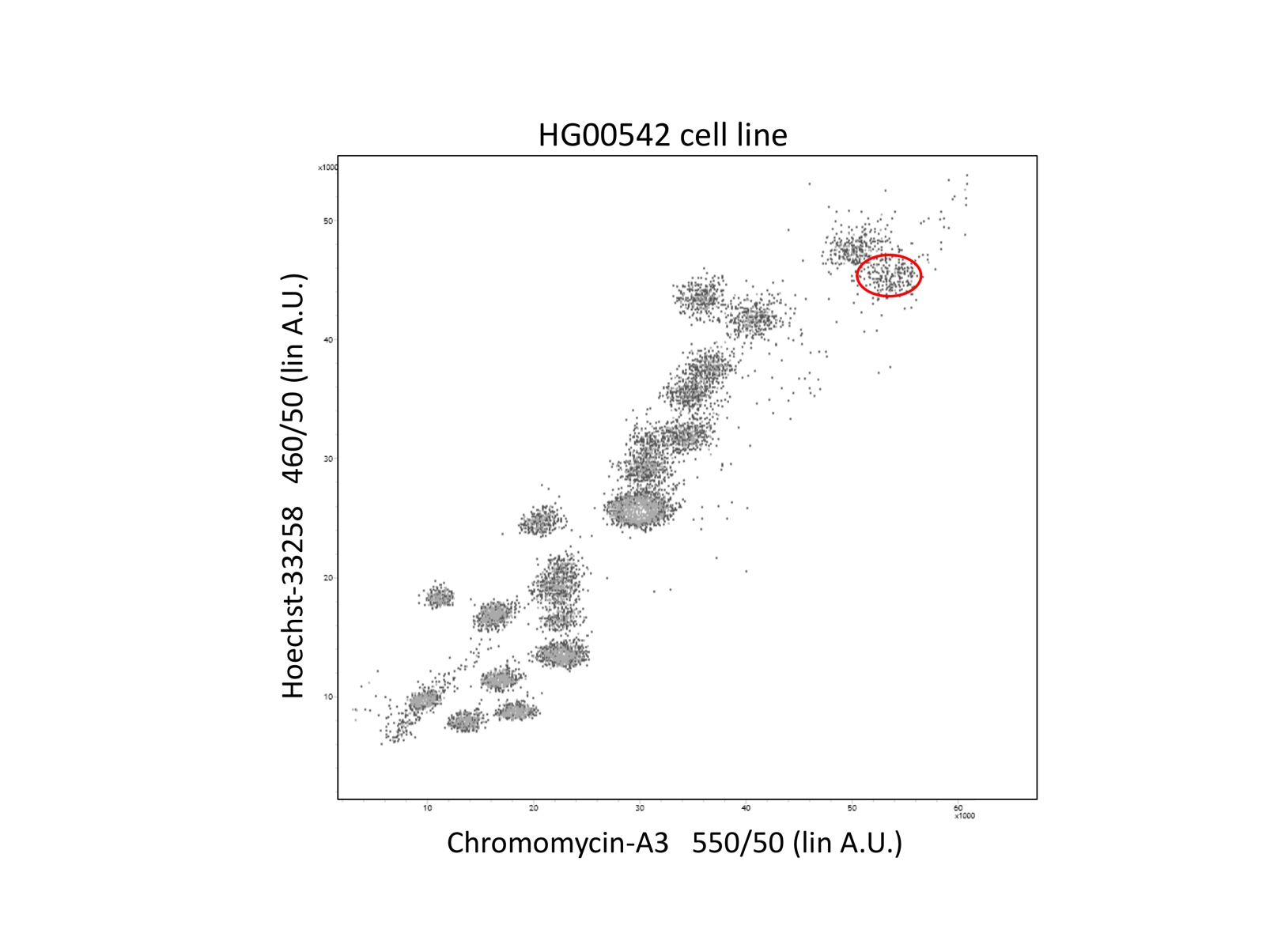


Supplementary figure 1: Flow karyogram of HG00542. The clusters correspond to different chromosomes. The red circle marks the selected cluster corresponding to chromosome 1 for flow-sorting.


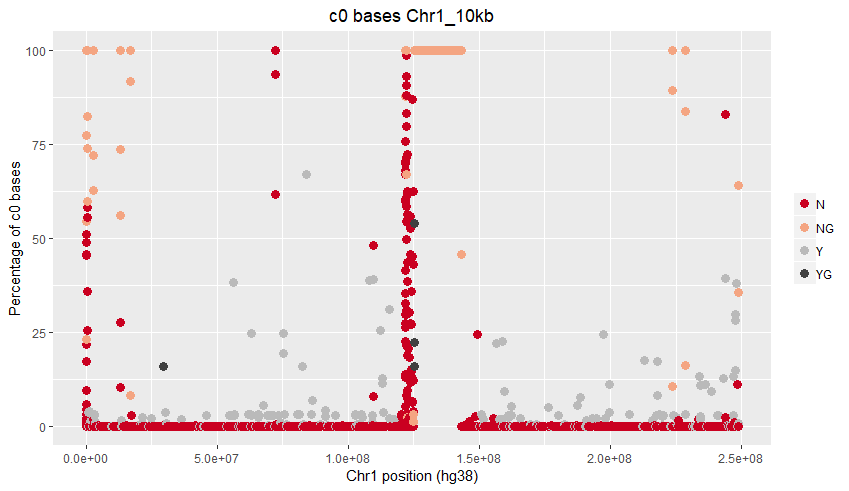
 Supplementary figure 2: Distribution of non covered bases in GRCh38 chromosome 1 (x-axis). Each dot represents a 10kb window, y-axis indicates the percentage of non covered bases per window. Colors show if the non covered bases can be explained by a defined gap in the GChg38 assembly or a SV detected by Assemblytics. N: windows intersecting no gaps, and no SV call; NG: windows intersecting gaps in GRCh38; Y: windows intersecting SV calls by Assemblytics; YG: windows intersecting SV calls and gaps.

Supplementary figure 3: Repeat content of the 6 most prominent repeat families. The number of annotated bases is very similar between the two assembly, showing good repeat resolution. The only exception are satellite sequences, which are better resolved in GRCh38

Supplementary figure 4: Overlap of SV-calls between Assemblytics and Sniffles.

Supplementary figure 5: Size distribution of SVs called by Assemblytics

Supplementary figure 6: Size distribution of SVs called by Sniffles

Supplementary figure 7: Size distribution of events called by the 1000G project.

Supplementary figure 8: Size distribution of SVs called by Audano et al.

Supplementary figure 9: Distribution of SVs by type called by Assemblytics

 Supplementary figure 10: Distribution of SVs by type called by Sniffles

 Supplementary figure 11: Distribution of SVs by type called by the 1000 genomes project.

 Supplementary figure 12: Distribution of SVs by type called by Audano et al. 2019.

| **Repeat element** | **GRCh38 bases** | **GRCh38**  **N annotations** | **HG00542_chr1 bases** | **HG00542_chr1**  **N entries** |
| --- | --- | --- | --- | --- |
| DNA? | 26591 | 218 | 26098 | 211 |
| DNA | 69648 | 460 | 68282 | 443 |
| DNA/Crypton | 2844 | 24 | 2977 | 24 |
| DNA/Crypton-A? | 1014 | 7 | 920 | 6 |
| DNA?/hAT? | 10601 | 61 | 10271 | 58 |
| DNA/hAT | 25438 | 133 | 25152 | 131 |
| DNA/hAT? | 7812 | 61 | 7163 | 59 |
| DNA/hAT-Ac? | 2838 | 19 | 2171 | 15 |
| DNA/hAT-Ac | 52221 | 274 | 51820 | 266 |
| DNA/hAT-Blackjack | 184189 | 1358 | 180850 | 1338 |
| DNA/hAT-Charlie | 3546372 | 20348 | 3461931 | 19924 |
| DNA/hAT-Tag1 | 35972 | 161 | 35737 | 163 |
| DNA?/hAT-Tip100? | 10533 | 40 | 10374 | 40 |
| DNA/hAT-Tip100? | 13027 | 54 | 12272 | 56 |
| DNA/hAT-Tip100 | 961889 | 5418 | 936867 | 5264 |
| DNA/Kolobok | 4124 | 22 | 3934 | 21 |
| DNA/Merlin | 3442 | 21 | 3475 | 21 |
| DNA/MULE-MuDR | 55139 | 178 | 54534 | 166 |
| DNA/MULE-MuDR? | 7897 | 48 | 7169 | 45 |
| DNA/PIF-Harbinger | 2006 | 15 | 2407 | 16 |
| DNA?/PiggyBac? | 1932 | 15 | 2178 | 19 |
| DNA/PiggyBac | 34605 | 158 | 34255 | 158 |
| DNA/TcMar? | 1867 | 11 | 1849 | 11 |
| DNA/TcMar-Mariner | 210209 | 1217 | 202006 | 1183 |
| DNA/TcMar-Pogo | 415 | 3 | 406 | 3 |
| DNA/TcMar-Tc1 | 9681 | 48 | 9428 | 48 |
| DNA/TcMar-Tc2 | 135604 | 717 | 133020 | 694 |
| DNA/TcMar-Tigger | 3039316 | 10791 | 2968878 | 10533 |
| LINE/CR1 | 1017377 | 6186 | 983052 | 6000 |
| LINE/Dong-R4 | 9304 | 44 | 8814 | 40 |
| LINE/I-Jockey | 843 | 8 | 926 | 10 |
| LINE/L1 | 39752285 | 91211 | 39007322 | 89426 |
| LINE/L1-Tx1 | 3886 | 12 | 3708 | 9 |
| LINE/L2 | 11776987 | 62636 | 11439435 | 61045 |
| LINE/Penelope | 3702 | 51 | 3758 | 52 |
| LINE/RTE-BovB | 68275 | 462 | 65297 | 456 |
| LINE/RTE-X | 235359 | 1089 | 229307 | 1058 |
| LTR | 113853 | 557 | 111432 | 544 |
| LTR? | 139383 | 698 | 133006 | 682 |
| LTR/ERV1? | 19923 | 115 | 18572 | 101 |
| LTR/ERV1 | 6827735 | 16851 | 6725085 | 16871 |
| LTR/ERVK | 764755 | 1049 | 749159 | 1058 |
| LTR/ERVL | 4663724 | 13612 | 4575158 | 13430 |
| LTR/ERVL? | 65008 | 265 | 62661 | 248 |
| LTR/ERVL-MaLR | 8258751 | 27705 | 8119090 | 27420 |
| LTR/Gypsy? | 113375 | 575 | 110656 | 561 |
| LTR/Gypsy | 269713 | 1268 | 260929 | 1217 |
| RC/Helitron | 21937 | 108 | 21480 | 104 |
| RC?/Helitron? | 6604 | 39 | 6745 | 40 |
| Retroposon/SVA | 826875 | 1606 | 789690 | 1540 |
| RNA | 9401 | 59 | 8699 | 56 |
| rRNA | 16744 | 174 | 16026 | 170 |
| Satellite | 33419 | 104 | 73534 | 348 |
| Satellite/acromeric | 69345 | 621 | 67889 | 579 |
| Satellite/centromeric | 6726363 | 40462 | 1662117 | 10288 |
| Satellite/telomeric | 15249 | 33 | 7748 | 12 |
| scRNA | 10543 | 118 | 10280 | 115 |
| SINE/5S-Deu-L2 | 18641 | 167 | 17774 | 155 |
| SINE/Alu | 26928414 | 104569 | 26014602 | 102278 |
| SINE/MIR | 9181107 | 68949 | 9016872 | 67924 |
| SINE/tRNA | 15080 | 132 | 15140 | 130 |
| SINE/tRNA-Deu | 3249 | 35 | 2495 | 23 |
| SINE/tRNA-RTE | 53624 | 425 | 50795 | 403 |
| snRNA | 37514 | 450 | 35148 | 424 |
| tRNA | 18628 | 286 | 26499 | 523 |
| Unknown | 68167 | 419 | 66209 | 406 |

Supplementary table 1: Cumulative length for all repeat elements for both GRCh38 chr 1 and HG00542 chr 1

|  | **Assemblytics** | **Sniffles** | **Overlap** |
| --- | --- | --- | --- |
| **SV calls** | 1325 | 405 | 940 |
| **SV calls spanning genes** | 779 | 353 | 230 |
| **SV calls in exonic region (n Genes)** | 117 (71) | 36 (24) | 8 (7) |
| **SV calls in intronic region (n Genes)** | 662 (387) | 317 (240) | 222 (175) |
|  |  |  |  |
| **Genes intersecting with SV calls (OMIM)** | 416 (216) | 180 (98) | 253 (137) |

Supplementary Table 2: Overview of number of SV calls across both datasets

|  | **Assemblytics** | **Sniffles** |
| --- | --- | --- |
| **Novel SV calls** | 685 | 306 |
| **Novel SV calls spanning genes** | 494 | 187 |
| **Novel SV calls in exonic region (n Genes)** | 51 (50) | 7 (6) |
| **Novel SV calls in intronic region (n Genes)** | 443 (300) | 180 (93) |
|  |  |  |
| **Novel Genes intersecting with SV calls (n genes OMIM)** | 322 (179) | 161 (96) |

Supplementary Table 3: Overview of previously undescribed SV candidates across both datasets

| Total SNPs | 284055 | 284055 |
| --- | --- | --- |
| AT | 10234 (3.60%) | 10755 (3.79%) |
| AC | 10882 (3.83%) | 13073 (4.60%) |
| AG | 43324 (15.25%) | 51711 (18.20%) |
| TA | 10755 (3.79%) | 10234 (3.60%) |
| TC | 44641 (15.72%) | 53109 (18.70%) |
| TG | 10600 (3.73%) | 13159 (4.63%) |
| CA | 13073 (4.60%) | 10882 (3.83%) |
| CT | 53109 (18.70%) | 44641 (15.72%) |
| CG | 11190 (3.94%) | 11377 (4.01%) |
| GA | 51711 (18.20%) | 43324 (15.25%) |
| GT | 13159 (4.63%) | 10600 (3.73%) |
| GC | 11377 (4.01%) | 11190 (3.94%) |
|  |  |  |
| Total GSNPs | 164111 | 164111 |
| AT | 4835 (2.95%) | 4837 (2.95%) |
| AC | 5650 (3.44%) | 6713 (4.09%) |
| AG | 27225 (16.59%) | 30940 (18.85%) |
| TA | 4837 (2.95%) | 4835 (2.95%) |
| TC | 27705 (16.88%) | 31511 (19.20%) |
| TG | 5570 (3.39%) | 6604 (4.02%) |
| CA | 6713 (4.09%) | 5650 (3.44%) |
| CT | 31511 (19.20%) | 27705 (16.88%) |
| CG | 6352 (3.87%) | 6169 (3.76%) |
| GA | 30940 (18.85%) | 27225 (16.59%) |
| GT | 6604 (4.02%) | 5570 (3.39%) |
| GC | 6169 (3.76%) | 6352 (3.87%) |

Supplementary table 4: Single nucleotide differences stratified by type. GSNPs denominate differences that are bound by 20 exact matches on either side.
